# Concordance between head and neck MRI and histopathology in detecting laryngeal subsite invasion among patients with laryngeal cancer

**DOI:** 10.1186/s40644-023-00618-y

**Published:** 2023-10-19

**Authors:** Issa Mohamad, Taher Abu Hejleh, Monther Qandeel, Maysa Al-Hussaini, Sami Koro, Ayat Taqash, Abdelatif Almousa, Fawzi Abuhijla, Ramiz Abuhijlih, Fatenah Ajlouni, Akram Al-Ibraheem, Dima Abu Laban, Tariq Hussein, Ebrahim Mayta, Wisam Al-Gargaz, Ali Hosni

**Affiliations:** 1 Department of Radiation Oncology, King Hussein Cancer Center, Amman, Jordan; 2 Department of Medical Oncology, King Hussein Cancer Center, Amman, Jordan; 3 Department of Diagnostic Radiology, King Hussein Cancer Center, Amman, Jordan; 4 Department of Pathology and Laboratory Medicine, King Hussein Cancer Center, Amman, Jordan; 5 Department of Radiation Oncology, Cleveland Clinic, Cleveland, OH USA; 6 Department of Biostatistics, King Hussein Cancer Center, Amman, Jordan; 7 Department of Nuclear Medicine, King Hussein Cancer Center, Amman, Jordan; 8 Department of Surgical Oncology, King Hussein Cancer Center, Amman, Jordan; 9 Department of Special Surgery, Jordan , University of Science and Technology, Irbid, Jordan; 10 Radiation Medicine Program, Princess Margaret Cancer Centre, University Health Network, University of Toronto, Toronto, ON Canada

**Keywords:** Laryngeal cancer, Total laryngectomy, Head and neck MRI, Histopathology

## Abstract

**Background:**

Accuracy of head and neck MRI (HN-MRI) in predicting tumor invasion of laryngeal site/subsites in patients with laryngeal cancer prior to laryngectomy is poorly evaluated in the literature. Therefore, we aim to evaluate the diagnostic value of HN-MRI in accurate pre-operative estimation of tumor invasion to laryngeal subsites in patients with laryngeal cancer.

**Methods:**

Patients with laryngeal cancer who underwent HN-MRI for cancer staging and underwent total laryngectomy between 2008 and 2021 were included. Sensitivity, specificity, positive predictive value, negative predictive value, and overall accuracy of HN-MRI in predicting tumor invasion of laryngeal subsites were calculated based on concordance between the HN-MRI and histopathological results.

**Results:**

One hundred and thirty-seven patients underwent total laryngectomy [primary: 82/137(60%), salvage 55/137(40%)]. The utilization of HN-MRI resulted in the downstaging of 16/137 (11.6%) patients and the upstaging of 8/137 (5.8%) patients. For the whole cohort, there was a significant discordance between HN-MRI and histopathology for T-category; out of 116 cT4a disease, 102(87.9%) were confirmed to have pT4a disease, and out of 17 cT3 disease, 9(52.9%) were confirmed to have pT3 disease, *p* < 0.001. The MRI overall diagnostic accuracy of predicting tumor invasion was 91%, 92%, 82%, 87%, 72%, 76%, 65% and 68% for base of tongue, arytenoid, vocal cord, posterior commissure, pre-epiglottic space, cricoid cartilage, inner thyroid cortex, and subglottis, respectively.

**Conclusions:**

In patients with laryngeal cancer undergoing total laryngectomy, HN-MRI demonstrates promising accuracy in predicting tumor invasion of specific laryngeal subsites (e.g., base of tongue). Our findings showed the potential of HN-MRI as a valuable tool for pre-operative planning and treatment decision-making in this patient population.

**Supplementary Information:**

The online version contains supplementary material available at 10.1186/s40644-023-00618-y.

## Background

The invasion of laryngeal tumor into the subsites of the larynx, such as penetration into the thyroid cartilage, is a significant factor in deciding whether to perform a total laryngectomy for laryngeal cancer patients versus functional preservation strategies [1–4]. The primary role of radiographic examination is to assess the depth of invasion of laryngeal tumor into adjacent structures, which might be under detected through endoscopic and physical examination only [5]. Head and neck magnetic resonance imaging (HN-MRI) is increasingly considered as one of used staging technique in laryngeal cancer, not only for accurate localization of laryngeal cancer, but also for precise evaluation of crucial anatomical subsites that impact treatment decision [6].

Advantages of HN-MRI imaging over CT encompass superior differentiation of soft tissues (i.e. extralaryngeal soft tissues invasion and base of tongue invasion), enabling the detection of subtle soft-tissue irregularities, enhanced discrimination between tumors and peritumoral inflammation, better assessment of laryngeal cartilage penetration, improved tumor outlining for targeted radiotherapy and transoral laser microsurgery, and enhanced diagnostic efficacy for identifying and precisely illustrating residual or recurring disease after treatment [6, 7], which impacts treatment selection and prognosis [8]. However, certain relative disadvantages of MR imaging, including lengthier scan times and the need for improved patient cooperation to counter motion artifacts, lower spatial resolution, and technical complexities related to air-tissue interfaces that impact the image quality of diffusion-weighted imaging (DWI) sequences [6].

Advanced imaging techniques beyond conventional HN-MRI improve the accuracy of staging laryngeal cancer [6]. The utilization of apparent diffusion coefficient (ADC), mainly in clinical setting, enables comprehensive assessment of the distinct characteristics associated with laryngeal cancer. Such imaging modalities can differentiate between tumor and peritumoral inflammation involving the thyroid cartilage, which was not possible with conventional HN-MRI [7]. This level of staging accuracy can lead to better treatment planning and outcomes of patients with laryngeal cancer [9]. Furthermore, continuous advancements in imaging technology and equipment offer the potential to obtain high-quality images of the entire larynx with isotropic resolution of 1 mm, free from any artifacts, in just a matter of minutes [10].

The purpose of this study was to assess the concordance between HN-MRI prediction of tumor invasion of laryngeal subsites only in patients with primary or recurrent laryngeal cancer prior to laryngectomy with post laryngectomy histopathological findings.

## Materials and methods

### Study population

The study was approved by the King Hussein Cancer Center (KHCC) Institutional Review Board (IRB) (No 21 KHCC 191). This is a retrospective analysis of adult patients with newly diagnosed or recurrent, non-metastatic squamous cell carcinoma (SCC) of the larynx who underwent preoperative HN-MRI staging and were treated with total laryngectomy at KHCC between 2009 and 2021 with available final pathological diagnosis. Within our study groups, no patients had received laser treatment or reconstructive surgery prior to undergoing total laryngectomy. Patients with recurrent laryngeal cancer were initially treated with radical radiotherapy were included in this study. Patients with histopathology other than SCC or contraindications to HN-MRI were excluded from this analysis. Data was gathered from medical records and included patient's demographics, radiographic, and pathological information. The TNM staging system was based on the eight edition of the American Join Committee on Cancer (AJCC) and Union of the International Cancer Control (UICC) TNM staging system [11].

### Diagnostic approach

The staging workup and pre-treatment evaluation included history and comprehensive physical examination. All patients underwent fiberoptic endoscopy, HN-MRI, and PET/CT. All cases were reviewed by the multidisciplinary H&N team to determine the best treatment approach.

### Imaging techniques and protocol

Prior to total laryngectomy, HN-MRI scans of patients with laryngeal cancer were thoroughly reviewed by at least 2 out of three experienced radiologists specializing in H&N imaging, each with at least over a decade of experience. These experts determined the extent of invasion in the laryngeal site/subsites and consistently reached a consensus that was documented in the HN-MRI report. The cases were equally distributed among the radiologists. There was no predetermined template for imaging review and the radiologists consistently identified sites/subsites of laryngeal invasion (See Supplementary table 1).

The images were obtained using either 1.5 T or 3 T MR scanners (3 T Philips Ingenia, 1.5 T Philips Ingenia and 1.5 T Siemens Avanto) using anterior surface neck coil and a slice thickness of 4 mm, intersection gap of 0.4 mm, field of view between 200 and 250 mm and matrix size between 125 × 125 to 270 × 250. The imaging protocol varied by the scanner but in general consisted of pre-contrast T1 images in the three orthogonal planes along with combination of axial and coronal T2 with fat saturation or T2 mDixon images as well as axial diffusion-weighted imaging (DWI) while the post contrast of T1 images with fat saturation or T1 mDixon sequences in three orthogonal planes. For DWI we use three different b values: 0, 500, and 1000 and standard field of view. Contrast-enhanced 3D GRE was administered intravenously to enhance the images, except for those with contraindications. The duration of the scanning process differs among these scanners, but it typically takes 35 min.

### Histopathology protocol

Grossing of formalin fixed laryngectomy specimens routinely consisted of handling and orientation of the laryngectomy specimen once received, with measurements of the specimen size. The outer surface was then inked and designation of color-code was noted in the sheet, followed by dissection and tumor localization including its relation to the true and false vocal cords, and aryepiglottis. The size of the tumor, and depth of invasion were measured. The location of the tumor in relation to important anatomical areas such as the thyroid and cricoid cartilage was documented. Measurements were taken and recorded to determine the distance between the tumor and the inked surgical margins. Subsequently, representative sections from the tumor, encompassing the maximum depth of invasion, the surrounding tissue and structures, as well as the margins, were evaluated.

### Data and statistical analysis

We assessed the concordance between HN-MRI and post laryngectomy histopathological examination of total laryngectomy specimens, specifically in determining the accuracy of invasion in different sites/subsites of the larynx. Histopathology post laryngectomy was considered the reference test for accurate determination of the extent of tumor invasion into laryngeal sites/subsites. The examination of the surgical specimens was conducted by experienced H&N pathologists who were blinded to the findings of the HN-MRI done before laryngectomy. The interpretations made by the radiologists based on the HN-MRI scans were subsequently compared to the histopathological reports. Each specific site or subsite in the larynx was classified as positive (indicating the presence of disease) or negative (indicating the absence of disease) based on the results obtained from both the HN-MRI and histopathological examinations. A dichotomous classification approach was employed to categorize the laryngeal sites/subsites using the findings derived from the HN-MRI scans and histopathological examination. Concordance between HN-MRI and histopathology was measured through calculating the sensitivity, specificity, positive predictive value, negative predictive value, and overall accuracy for all laryngeal sites/subsites [12].

Descriptive analysis of patients’ information was performed. Categorical variables, such as gender, smoking and other factors were presented as counts and percentages. The mean (standard deviation) and/or median (range) were calculated for the continuous variables. Fisher's exact test was used to determine if there were nonrandom associations between primary versus salvage cohorts, a significance criterion of *p* ≤ 0.05 was used in the analysis. All analyses were performed using SAS version 9.4 (SAS Institute Inc, Cary, NC).

## Results

### Patient and tumor characteristics

A total of 137 patients with laryngeal cancer were identified in this study. The median (range) age of the patients was 60 (37–96) years. Most of the patients were males (93.4%). The majority were smokers (95%) with only (10%) reporting history of drinking alcohol. Of the 137 patients, 82 (60%) underwent staging HN-MRI before total laryngectomy for primary laryngeal cancer, and 55 (40%) patients had re-staging HN-MRI for recurrent laryngeal cancer before salvage laryngectomy. The final histopathological results showed that the majority of patients 98 (71.5%) had overlapping laryngeal tumors, while 32 (23.4%) had glottic tumors, and 7 (5.1%) had supraglottic tumors as shown in Table 1.

**Table 1:** Patients, tumor and treatment characteristics

Variable	Value	Number (%)	Cohort N = 137		*P*-value
			Primary 82(60%)	Salvage 55(40%)	
Age, median (range)	-	60(37–96)	59(45–96)	61(37–85)	0.13
Gender	Female	9(6.6%)	7(8.5%)	2(3.6%)	0.31
	Male	128(93.4%)	75(91.5%)	53(96.4%)	
History of smoking	Yes	130(94.9%)	77(94%)	53(96.4%)	0.52
	No	2(1.5%)	2(2.5%)	-	
	Unknown	5(3.6%)	3(3.5%)	2(3.6%)	
History of drinking	Yes	14(10.2%)	11(13.5%)	3(5.5%)	0.18
	No	115(84%)	66(80.5%)	49(89%)	
	Unknown	8(5.8%)	5(6%)	3(5.5%)	
Primary tumor subsite	Glottis	32(23.4%)	15(18.3%)	17(30.9%)	0.12
	Supraglottis	7(5.1%)	3(3.7%)	4(7.3%)	
	Overlapping	98(71.5%)	64(78%)	34(61.8%)	

### Radiographic-pathological correlation of T-category

Among the whole cohort (i.e. patients who underwent total laryngectomy whether for primary or recurrent laryngeal cancer, *n* = 137), there was a significant discordant between HN-MRI and histopathology for T-category (*p* < 0.001) (Table 2). Out of 116 patients with cT4a disease: 102 (87.9%) were confirmed to have pT4a disease, of 17 patients with cT3 disease: 13 (52.9%) were confirmed to have pT3 disease, of 2 patients with cT2 disease: 2 (100%) were confirmed to have pT2 disease, and out of 2 patients with cT1 disease 0 (0%) were confirmed to have pT1 disease.

**Table 2:** Concordant/discordant agreement between staging HN-MRI and histopathology for T-category

	Patients (N)	Pathological T-Category					
		pT0	pT1	pT2	pT3	pT4a	*p*-value
HN-MRI T-Category All patients	137	2/137	-	3/137	23/137	109/137	
cT1	2/137	-	-	-	1/2(50%)	1/2(50%)	0.001
cT2	2/137	-	-	2/2(100%)	-	-	
cT3	17/137	1/17(5.9%)		1/17(5.9%)	9/17(52.9%)	6/17(35.3%)	
cT4a	116/137	1/116(0.9%)	-	-	13/116(11.2%)	102/116(87.9%)	
HN-MRI T-Category (Primary laryngectomy)		pT0	pT1	pT2	pT3	pT4	p-value
Total	82	-	-	1/82	12/82	69/100	
cT1	-	-	-	-	-	-	0.004
cT2	-	-	-	-	-	-	
cT3	4/82	-	-	1/4(25%)	2/4(50%)	1/4(25%)	
cT4a	78/82	-	-	-	10/78(12.8%)	68/78(87.2%)	
HN-MRI T-Category (Salvage laryngectomy)		rpT0	rpT1	rpT2	rpT3	rpT4a	p-value
Total	55	2/55	-	2/55	11/55	40/55	
cT1	2/55	-	-	-	1/2(50%)	1/2(50%)	0.001
cT2	2/55	-	-	2/2(100%)	-	-	
cT3	13/55	1/13(7.7%)	-	-	7/13(53.8%)	5/13(38.5%)	
cT4a	38/55	1/38(2.6%)	-	-	3/38(7.9%)	34/38(89.5%)	

*Abbreviations*: *T* Tumor, *HN* Head and neck

In a subgroup of patients who had primary laryngeal cancer (*n* = 82), there was a significant discordant between HN-MRI and histopathology for T-category (*p* < 0.004) (Table 2). Out of seventy eight cT4a disease: 68 (87.2%) were confirmed to have pT4a disease, and of four cT3 disease: 2 (50%) were confirmed to have pT3 disease.

In a subgroup of patients who had recurrent laryngeal cancer (*n* = 55), there was a significant disagreement between HN-MRI and histopathology for T-category (*p* < 0.001) (Table 2), out of thirty eight cT4a disease: 34 (89.5%) were confirmed to have pT4a disease, of thirteen cT3 disease: 7 (53.8%) were confirmed to have pT3 disease, of two cT2 disease: 2 (100%) were confirmed to have pT2 disease, and out of two cT1 disease: 0 (0%) were confirmed to have pT1 disease.

**Fig. 1:**
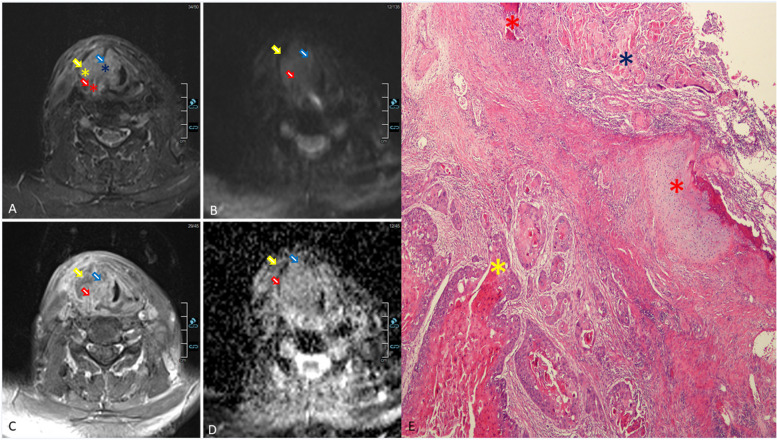
Radiological pathological correlation. Axial STIR (**A**), Axial DWI (**B**), axial post-contrast fat-saturated T1 (**C**), and axial ADC (**D**) represents HN-MRI imaging set for patient with laryngeal cancer prior to laryngectomy at the level of the glottis showing a lobulated, heterogeneous right glottic mass (blue asterisk/arrow) that invades the right thyroid cartilage lamina (red asterisk/arrow) resulting in extralaryngeal extension (yellow asterisk/arrow). **E** Microscopic section from the moderately differentiated squamous cell carcinoma invading through thyroid cartilage, and extending to the adjacent soft tissue (H&E, X4). Areas correlated between MRI and histopathology are marked by colored asterisks; red; thyroid cartilage, blue; tumor in the larynx, yellow; tumor in extralaryngeal soft tissue

Figure 1 displays concordant example between HN-MRI and histopathology results.

**Fig. 2:**
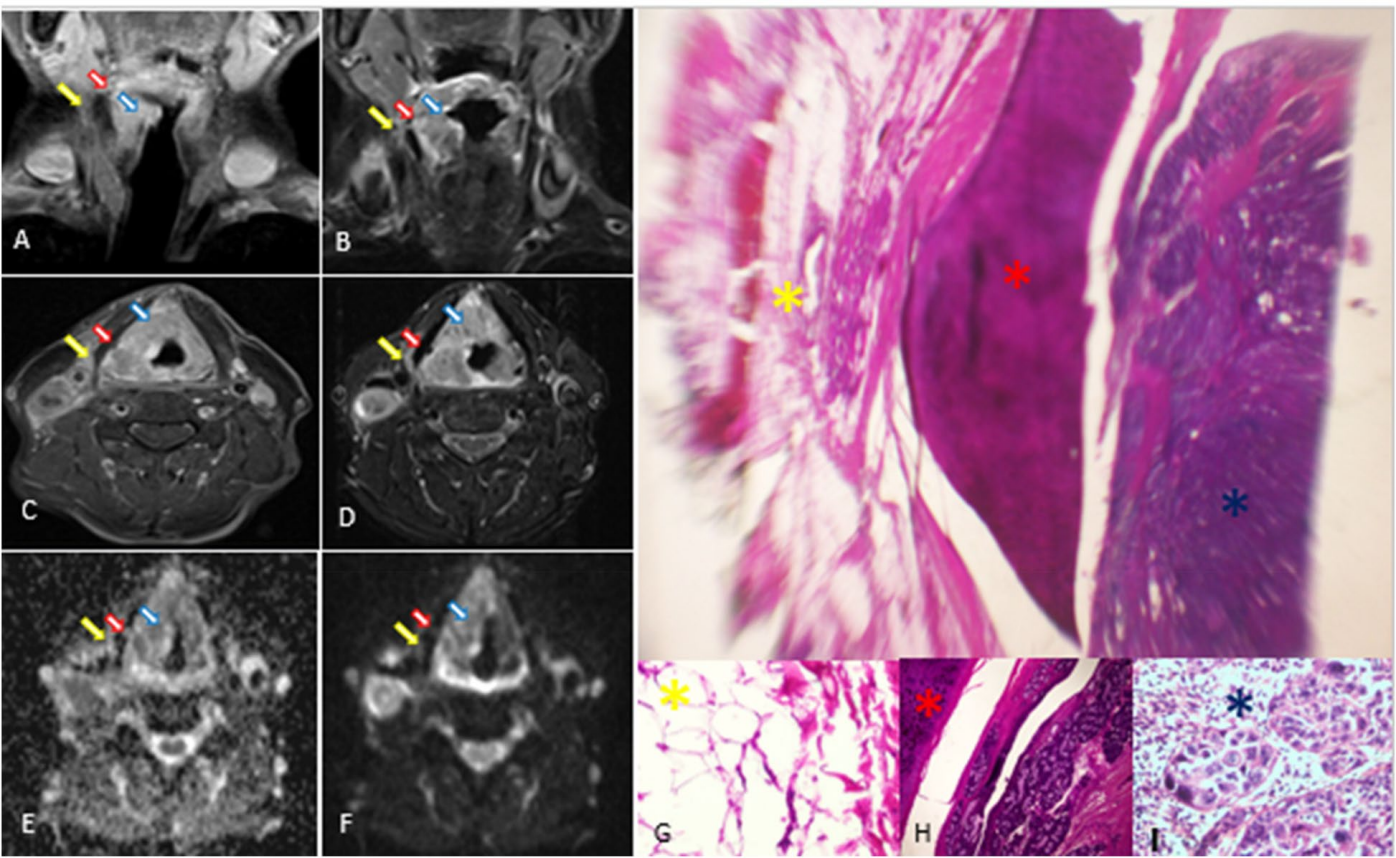
Radiological pathological correlation. Coronal T1weighted post-contrast fat-saturated (**A**), Coronal STIR (**B**), Axial T1weighted post-contrast fat-saturated (**C**), axial STIR (**D**), axial DWI (**E**), and axial ADC (**F**) represents HN-MRI imaging set for patient with laryngeal cancer prior to laryngectomy showing right laryngeal mass at glottis level with supraglottic extension (blue arrow), with focal STIR hyperintense signal and contrast enhancement just external to the right thyroid cartilage lamina (yellow arrow) without thyroid cartilage penetration (red arrow). Scanning power microscopic section from the moderately differentiated squamous cell carcinoma approaching but not invading thyroid cartilage, and with benign adjacent soft tissue (H&E, X2.5). Areas correlated between MRI and histopathology are marked by colored asterisks; yellow (**G**); Extralaryngeal soft tissue, red (**H**); thyroid cartilage, and blue (**I**); tumor in the larynx

Figure 2 displays discordant example between HN-MRI and histopathology results.

For the whole cohort, the utilization of HN-MRI resulted in the downstaging of 16/137 (11.6%) patients and the upstaging of 8/137 (5.8%) patients. For primary laryngectomy vs salvage laryngectomy cohort, the utilization of HN-MRI resulted in the downstaging of 11/82 (13.4%) vs 5/55 (9.1%) patients and the upstaging of 1/82 (1.2%) vs 7/55 (12.7%) patients, respectively.

The results of a sub-analysis, which compared patients who underwent the DWI HN-MRI sequence with those who did not, demonstrated an increased probability of detecting full-thickness thyroid cartilage destruction (*p* = 0.049) and extension of tumor into subglottis (*p* = 0.014) among patients who underwent DWI imaging as shown in the Supplementary table 4.

### Diagnostic performance of laryngeal MRI

As shown in Table 3, for the whole cohort, the MRI's ability to predict tumor extension into laryngeal subsites varied across different tumor locations. The accuracy was 91%, 92%, 82%, 87%, 72%, 76%, 65% and 68% for base of tongue, arytenoid, vocal cord, posterior commissure, pre-epiglottic space, cricoid cartilage, inner thyroid cortex, and subglottis subsites extension, respectively. The details of sensitivity, specificity, negative predictive value, positive predictive value and accuracy of HN-MRI of predicting tumor extension into laryngeal subsites compared with histopathological findings for the whole cohorts is summarized in Table 3, for the primary and salvage cohorts, this data is provided in Supplementary tables 2 and 3, and for patients who underwent the DWI sequence and conventional HN-MRI, it can be found in Supplementary tables 5 and 6.

**Table 3:** Shows sensitivity, specificity, negative predictive value, positive predictive value, and accuracy of HN-MRI in predicting the extension of tumors into laryngeal subsites, compared with the results of histopathological examinations for the whole cohorts

**Tumor extension to**	**Pathologic involvement**	**Radiologic involvement**	**Sensitivity (%)**	**Specificity (**%)	**Positive predictive value (**%)	**Negative predictive value (**%)	**Overall accuracy (**%)
Supraglottis	104	116	87	76	95	52	85
Supra and infra-hyoid epiglottis	17	51	22	93	65	67	66
Aryepiglottic folds, laryngeal aspect	32	78	33	90	81	50	58
Arytenoids	3	12	17	99	67	93	92
False vocal cords	26	81	28	95	88	48	55
True vocal cord/Glottis	110	115	87	55	91	44	82
Paraglottic space	24	88	20	88	75	38	45
Pre-eiglottic space	20	50	32	95	80	71	72
Inner cortex of thyroid cartilage	62	82	59	75	77	55	65
Anterior commissures	12	67	12	94	67	53	54
Posterior commissures	1	17	0	99	0	88	87
Subglottis	46	68	51	84	76	64	68
Cricoid cartilage	35	44	52	87	66	79	76
Full-thickness thyroid cartilage	84	86	74	61	76	58	69
Extralaryngeal soft tissue of the neck	53	72	56	80	75	62	67
Base of tongue	11	15	47	97	64	94	91

## Discussion

In the current study, HN-MRI was found to be approximately 87% accurate in correctly diagnosing a T4a laryngeal tumor, which was consistent with the results of post laryngectomy histopathological examination. However, the study also indicated that there was variability in the accuracy of HN-MRI in identifying the extent of the tumor into adjacent anatomical subsites, regardless of whether the laryngeal tumor was primary or recurrent. Across the entire group of patients, HN-MRI was most accurate in identifying tumor invasion into the arytenoid and base of the tongue (more than 90% accuracy), while the lowest accuracy was in detecting tumor extension into the paraglottic space (45%). Additionally, HN-MRI was observed to be more specific than sensitive in detecting tumor extension into laryngeal subsites.

Accurate identification of the anatomic subsites involved in the larynx is crucial, and the use of the most recent staging edition of AJCC/TNM nomenclature impacts treatment selection and prognosis [2, 13–15]. Clinical evaluation is superior to HN-MRI in assessing the extent of early stage laryngeal tumors on the mucosal surface [14], whereas HN-MRI excels in assessing the deeper submucosal extent of the tumor, including involvement of the base of the tongue [6]. For instance, the involvement of the paraglottic and pre-epiglottic spaces may necessitate a change in treatment from definitive radiotherapy to concurrent chemoradiation (CRT), while cartilage penetration may require a change in treatment approach from functional preservation to total laryngectomy [2, 16]. In this study, there was a significant difference between the HN-MRI cT-category and the pT-category findings (*p* < 0.05) which led to over- or under-staging of cT-category (i.e. 12% of patients underwent unnecessary total laryngectomy due to over-staging of HN-MRI), which confirm the observation found by Kim and Nix [17, 18]. In the current study, the accuracy of HN-MRI in detecting supraglottic laryngeal cancer was 85%, which was higher than the accuracy found by Kim et al. (75%) [17]. This discrepancy may be due to the radiologists' cautious reporting approach that focused on the critical decision-making tumor extension to the laryngeal subsites and false interpretation caused by peritumoral inflammation [19]. To enhance the accuracy of HN-MRI, it's crucial to establish a new set of guidelines "laryngeal-RADs," which may standardize the interpretation of MRI results for laryngeal cancer and provide a clear implication of decision-making directed treatment approach [20].

Regarding paraglottic space infiltration, our study showed low sensitivity (20%) and high specificity (88%), compared with other studies that reported higher sensitivity (93–95%), but lower specificity (50–76%) [21, 22]. Detecting pre-epiglottic space infiltration is also difficult, as our study showed a sensitivity of only 32% but a high specificity of 95%. Other studies have reported higher sensitivity (100%) but lower specificity (85%) in this regard [21]. The challenge lies in accurately determining the boundary of primary tumor from the surrounding peri-tumoral inflammation, edema, and fibrosis [22]. The paraglottic space is best assessed on axial and coronal images and HN-MRI is superior to CT for assessment of paraglottic space [21, 22]. The pre-epiglottic space is best visible on axial and sagittal images [22]. These spaces are mostly composed of fat, which is bright on T1 and T2 sequences and shows no or only minimal contrast enhancement [22]. In contrast, laryngeal cancer show intermediate signal intensity on T1, T2, and STIR and moderate post contrast enhancement [22]. Compared to the tumor itself, peritumoral inflammation tends to show higher T2 signal, more intense contrast enhancement and less diffusion restriction [6, 7]. Relying more on DWI in such cases could have improved the concordance rates.

Regarding thyroid cartilage invasion, a study conducted by Taha et al., had reported higher sensitivity, specificity, positive predictive value, and negative predictive value of HN-MRI in detecting inner thyroid lamina invasion (93%, 82%, 88%, and 90%) [23] compared to our study's findings (59%, 75%, 77%, and 55%, respectively). Also, Taha et al., had reported a higher sensitivity, specificity, positive predictive value, and negative predictive value of 85% for full thickness thyroid cartilage invasion [23] compared to our study's findings (74%, 61%, 79%, and 79%, respectively). Li et al., had reported that neck CT scans had a lower sensitivity, specificity, positive predictive value, and negative predictive value (57%, 86%, 65%, and 81%, respectively) compared with HN-MRI ( 94%, 87%, 78%, and 97%, respectively) [24]. Our study showed lower HN-MRI sensitivity, specificity, positive predictive value, and negative predictive value of (74%, 61%, 76%, and 58%, respectively) compared to Li et al., study. Perhaps, relying on multiparametric MRI assessment could improve the agreement rates in our study, which can differentiate between normal and ossified cartilage, peritumoral inflammation, and tumoral invasion [6, 7]. Neck CT scan cannot reliably make that distinction between the normal and ossified cartilage, sclerosis due to tumor invasion and sclerosis due to peritumoral inflammation [7].

Differentiating between cT3 and cT4 holds critical significance for the management and prognosis of laryngeal cancer patients [2, 25]. Tumors labeled as cT4a due to cartilage penetration exhibit heightened local recurrence risk post functional preservation. The VA-larynx randomized clinical trial revealed that salvage laryngectomy was necessary for 44% of Stage IV laryngeal cancer patients compared to 29% of Stage III (*P* = 0.048) and 56% of T4 tumors (those with cartilage penetration) vs 29% of smaller primary tumors (*P* = 0.001) [25]. Notably, RTOG 91–11 excluded cT4 patients due to the increased local failure risk observed in the VA-RCT [2, 25]. Within our clinical practice guidelines, we recommend total laryngectomy for patients with T4 disease due to cartilage penetration, substantial bulky T3 tumors with a non-functioning larynx, and as a salvage option following recurrence or persistent disease post functional preservation treatment [15]. Our study showed that 10/78 (12.8%) patients categorized as cT4, and without prior laryngeal treatment, underwent unnecessary primary laryngectomy and 1/4 (25%) categorized as cT3 disease found to have cT2 disease after laryngectomy who was candidate for functional preservation approach.

Limitations of this study include its retrospective nature. The study has inherent bias because we only reviewed cases that went to laryngectomy and missing true overall population sensitivity/specificity/accuracy in all comers, furthermore, the study included only those who had disease that was determined to be surgical. Incomplete radiology reports can also limit the study's findings as not all laryngeal subsites were reported. This means that the study may miss some important details that could affect the interpretation of the results. In addition, variability of MR technique and different MR scanners used for imaging the patients may also be contributing the study limitation. Furthermore, the lack of use of DWI/ADC in a substantial proportion of patients. However, our study reports on a relatively large cohort of a homogenous group of patients who received the same treatment protocol. Imaging the larynx with MRI is challenging mainly because of the long acquisition time and motion artifact induced by breathing, swallowing, and vessel pulsation but this has improved with newer techniques, including parallel imaging, and equipment [26]. The advancement in technology have also led to significant improvements in the image quality and spatial resolution, which can lead to more reliable assessment of cartilage invasion for example [7]. The addition of DWI to the magnetic resonance protocol has the potential to increase the specificity of the diagnosis for cartilage involvement and for discrimination of peritumoral edema from neoplastic tissue when assessing paraglottic space invasion [23, 27]. Dynamic contrast-enhanced magnetic resonance imaging (DCE-MRI) can help better differentiate between recurrent malignant tumors after CRT and changes caused by CRT [28]. DCE-MRI metrics can also predict response to CRT and potentially guide treatment [29–31]. Combined, parallel imaging and other technique have significantly improved the imaging acquisition times so that high temporal resolution cine MR imaging of the larynx is now possible at 3 T, which can demonstrate and quantify the intrafractional HN tumor motion and can help improve radiation planning and delivery [32, 33].

## Conclusions

In patients with laryngeal cancer undergoing total laryngectomy, HN-MRI demonstrates promising accuracy in predicting tumor invasion of specific laryngeal subsites (e.g., base of tongue). Our findings showed the potential of HN-MRI as a valuable tool for pre-operative planning and treatment decision-making in this patient population.

### Supplementary Information

**Additional file 1: Supplementary table 1.** Radiologists demographics.

**Additional file 2: Supplementary table 2.** Sensitivity, specificity, negative predictive value, positive predictive value, and accuracy of HN-MRI in predicting the extension of tumors into laryngeal subsites, compared with the results of histopathological examinations for patient who underwent primary laryngectomy.

**Additional file 3: Supplementary table 3.** Sensitivity, specificity, negative predictive value, positive predictive value, and accuracy of HN-MRI in predicting the extension of tumors into laryngeal subsites, compared with the results of histopathological examinations for patient who underwent salvage laryngectomy.

**Additional file 4: Supplementary table 4.** provides data indicating the effectiveness of DWI sequences in HN-MRI for identifying the spread of tumors into specific laryngeal subsites.

**Additional file 5: Supplementary table 5.** presents the sensitivity, specificity, negative predictive value, positive predictive value, and accuracy of the DWI HN-MRI sequence when predicting tumor extension into laryngeal subsites, in comparison to the findings from histopathological assessments for patients who underwent total laryngectomy.

**Additional file 6: Supplementary table 6.** presents the sensitivity, specificity, negative predictive value, positive predictive value, and accuracy of the conventional HN-MRI when predicting tumor extension into laryngeal subsites, in comparison to the findings from histopathological assessments for patients who underwent total laryngectomy.

## Data Availability

Data is available upon request.

## Abbreviations

HN-MRI: Head and neck MRI
ADC: Apparent diffusion coefficient
DCE: Dynamic contrast-enhanced
KHCC: King Hussein Cancer Center
IRB: Institutional Review Board
SCC: Squamous cell carcinoma
AJCC: American Join Committee on Cancer
UICC: Union of the International Cancer Control
DWI: Diffusion-weighted imaging
H&N: Head and neck
