# Supplementary material for: Concordance between head and neck MRI and histopathology in detecting laryngeal subsite invasion among patients with laryngeal cancer
Source: Cancer Imaging. 2023 Oct 19;23:99. doi: 10.1186/s40644-023-00618-y (PMC10585883; doi:10.1186/s40644-023-00618-y)
Supplement: Supplementary file 1 — Additional file 1: Supplementary table 1. Radiologists demographics. [file 40644_2023_618_MOESM1_ESM.docx]

Supplementary table 1: Radiologists demographics

|  | Radiologist “1” | Radiologist “2” | Radiologist “3” |
| --- | --- | --- | --- |
| Post-graduate training   - No. of years/Location | - 4 years residency/ Jordanian board (1998/Jordan)  - FRCR (2006/UK) | - 4 years residency/ Jordanian board (2003/Jordan)  - FRCR (2007/UK) | - 5 years residency/American board of radiology (2010/USA)  - Pediatric fellowship (2011/USA)  - Neuroradiology fellowship (2016/USA) |
| Independent HN practice   - No. of years until 2008 - No of years until 2021 - Location | - 10 years  - 23 years  - KHCC | - 5 years  - 18 years  - KHCC | - 0 year  -10 years  - 9 years/KHCC, 1 year/USA |
| Number of reports per radiologist for the study population | 55 | 48 | 34 |
