# Supplementary material for: Concordance between head and neck MRI and histopathology in detecting laryngeal subsite invasion among patients with laryngeal cancer
Source: Cancer Imaging. 2023 Oct 19;23:99. doi: 10.1186/s40644-023-00618-y (PMC10585883; doi:10.1186/s40644-023-00618-y)
Supplement: Supplementary file 2 — Additional file 2: Supplementary table 2. Sensitivity, specificity, negative predictive value, positive predictive value, and accuracy of HN-MRI in predicting the extension of tumors into laryngeal subsites, compared with the results of histopathological examinations for patient who underwent primary laryngectomy. [file 40644_2023_618_MOESM2_ESM.docx]

| **Tumor extension to** | **Pathologic involvement** | **Radiologic involvement** | **Sensitivity (%)** | **Specificity (**%) | **Positive predictive value (**%) | **Negative predictive value (**%) | **Overall accuracy (**%) |
| --- | --- | --- | --- | --- | --- | --- | --- |
| Supraglottis | 67 | 75 | 89 | 100 | 100 | 47 | 90 |
| Supra and infra-hyoid epiglottis | 9 | 39 | 21 | 98 | 89 | 58 | 61 |
| Aryepiglottic folds, laryngeal aspect | 22 | 50 | 36 | 88 | 82 | 47 | 56 |
| Arytenoids | 2 | 8 | 25 | 100 | 100 | 93 | 93 |
| False vocal cords | 18 | 56 | 29 | 92 | 89 | 38 | 49 |
| True vocal cord/Glottis | 70 | 72 | 89 | 40 | 91 | 33 | 83 |
| Paraglottic space | 15 | 64 | 19 | 83 | 80 | 22 | 33 |
| Pre-eiglottic space | 14 | 36 | 36 | 98 | 93 | 66 | 71 |
| Inner cortex of thyroid cartilage | 42 | 63 | 56 | 63 | 83 | 30 | 57 |
| Anterior commissures | 8 | 46 | 13 | 94 | 75 | 46 | 49 |
| Posterior commissures | 0 | 8 | 0 | 100 | 0 | 90 | 90 |
| Subglottis | 22 | 42 | 38 | 83 | 68 | 55 | 59 |
| Cricoid cartilage | 18 | 26 | 50 | 91 | 72 | 80 | 78 |
| Full-thickness thyroid cartilage | 57 | 64 | 75 | 50 | 81 | 36 | 70 |
| Extralaryngeal soft tissue of the neck | 39 | 52 | 58 | 70 | 77 | 49 | 62 |
| Base of tongue | 8 | 10 | 50 | 96 | 63 | 93 | 90 |

Supplementary table 2: Sensitivity, specificity, negative predictive value, positive predictive value, and accuracy of HN-MRI in predicting the extension of tumors into laryngeal subsites, compared with the results of histopathological examinations for patient who underwent primary laryngectomy
