# Supplementary material for: Concordance between head and neck MRI and histopathology in detecting laryngeal subsite invasion among patients with laryngeal cancer
Source: Cancer Imaging. 2023 Oct 19;23:99. doi: 10.1186/s40644-023-00618-y (PMC10585883; doi:10.1186/s40644-023-00618-y)
Supplement: Supplementary file 3 — Additional file 3: Supplementary table 3. Sensitivity, specificity, negative predictive value, positive predictive value, and accuracy of HN-MRI in predicting the extension of tumors into laryngeal subsites, compared with the results of histopathological examinations for patient who underwent salvage laryngectomy. [file 40644_2023_618_MOESM3_ESM.docx]

| **Tumor extension to** | **Pathologic involvement** | **Radiologic involvement** | **Sensitivity (%)** | **Specificity (**%) | **Positive predictive value (**%) | **Negative predictive value (**%) | **Overall accuracy (**%) |
| --- | --- | --- | --- | --- | --- | --- | --- |
| Supraglottis | 37 | 41 | 83 | 64 | 87 | 56 | 78 |
| Supra and infra-hyoid epiglottis | 8 | 12 | 25 | 88 | 38 | 81 | 75 |
| Aryepiglottic folds, laryngeal aspect | 10 | 28 | 29 | 93 | 80 | 56 | 60 |
| Arytenoids | 1 | 4 | 0 | 98 | 0 | 93 | 91 |
| False vocal cords | 8 | 25 | 28 | 97 | 88 | 62 | 65 |
| True vocal cord/Glottis | 40 | 43 | 84 | 67 | 90 | 53 | 80 |
| Paraglottic space | 9 | 24 | 25 | 90 | 67 | 61 | 62 |
| Pre-eiglottic space | 6 | 14 | 21 | 93 | 50 | 78 | 75 |
| Inner cortex of thyroid cartilage | 20 | 19 | 68 | 81 | 65 | 83 | 76 |
| Anterior commissures | 4 | 21 | 10 | 94 | 50 | 63 | 62 |
| Posterior commissures | 1 | 9 | 0 | 98 | 0 | 83 | 82 |
| Subglottis | 24 | 26 | 77 | 86 | 83 | 81 | 82 |
| Cricoid cartilage | 17 | 18 | 56 | 81 | 59 | 79 | 73 |
| Full-thickness thyroid cartilage | 27 | 22 | 73 | 67 | 59 | 79 | 69 |
| Extralaryngeal soft tissue of the neck | 14 | 20 | 50 | 89 | 71 | 76 | 75 |
| Base of tongue | 3 | 5 | 40 | 98 | 67 | 94 | 93 |

Supplementary table 3: Sensitivity, specificity, negative predictive value, positive predictive value, and accuracy of HN-MRI in predicting the extension of tumors into laryngeal subsites, compared with the results of histopathological examinations for patient who underwent salvage laryngectomy
