# Supplementary material for: Concordance between head and neck MRI and histopathology in detecting laryngeal subsite invasion among patients with laryngeal cancer
Source: Cancer Imaging. 2023 Oct 19;23:99. doi: 10.1186/s40644-023-00618-y (PMC10585883; doi:10.1186/s40644-023-00618-y)
Supplement: Supplementary file 4 — Additional file 4: Supplementary table 4. provides data indicating the effectiveness of DWI sequences in HN-MRI for identifying the spread of tumors into specific laryngeal subsites. [file 40644_2023_618_MOESM4_ESM.doc]

Supplementary table 4 provides data indicating the effectiveness of DWI sequences in HN-MRI for identifying the spread of tumors into specific laryngeal subsites.

| Subsite involvement | Value | Total (n=) | DWI | | P-value |
| --- | --- | --- | --- | --- | --- |
|  |  |  | Performed (n/%) | Not performed (n/%) |  |
| Supraglottis | Not involved | 31 | 20(20.8%) | 11(26.8%) | 0.442 |
|  | Involved | 106 | 76(79.2%) | 30(73.2%) |  |
| Supra and infra-hyoid epiglottis | Not involved | 120 | 86(89.6%) | 34(82.9%) | 0.279 |
|  | Involved | 17 | 10(10.4%) | 7(17.1%) |  |
| Aryepiglottic folds, laryngeal aspect | Not involved | 105 | 73(76.0%) | 32(78.0%) | 0.799 |
|  | Involved | 32 | 23(24.0%) | 9(22.0%) |  |
| Arytenoids | Not involved | 134 | 94(97.9%) | 40(97.6%) | 1.000 |
|  | Involved | 3 | 2(2.1%) | 1(2.4%) |  |
| False vocal cords | Not involved | 111 | 77(80.2%) | 34(82.9%) | 0.710 |
|  | Involved | 26 | 19(19.8%) | 7(17.1%) |  |
| True vocal cord/Glottis | Not involved | 27 | 19(19.8%) | 8(19.5%) | 0.970 |
|  | Involved | 110 | 77(80.2%) | 33(80.5%) |  |
| Paraglottic space | Not involved | 113 | 77(80.2%) | 36(87.8%) | 0.335 |
|  | Involved | 24 | 19(19.8%) | 5(12.2%) |  |
| Pre-epiglottic space | Not involved | 117 | 81(84.4%) | 36(87.8%) | 0.793 |
|  | Involved | 20 | 15(15.6%) | 5(12.2%) |  |
| Inner cortex of thyroid cartilage | Not involved | 75 | 48(50.0%) | 27(65.9%) | 0.088 |
|  | Involved | 62 | 48(50.0%) | 14(34.1%) |  |
| Anterior commissures | Not involved | 125 | 88(91.7%) | 37(90.2%) | 0.751 |
|  | Involved | 12 | 8(8.3%) | 4(9.8%) |  |
| Posterior commissures | Not involved | 136 | 96(100%) | 40(97.6%) | 0.299 |
|  | Yes | 1 |  | 1(2.4%) |  |
| Subglottis | Not involved | 91 | 70(72.9%) | 21(51.2%) | **0.014** |
|  | Involved | 46 | 26(27.1%) | 20(48.8%) |  |
| Cricoid cartilage | Not involved | 102 | 73(76.0%) | 29(70.7%) | 0.514 |
|  | Involved | 35 | 23(24.0%) | 12(29.3%) |  |
| Full-thickness thyroid cartilage | Not involved | 53 | 32(33.3%) | 21(51.2%) | **0.049** |
|  | Involved | 84 | 64(66.7%) | 20(48.8%) |  |
| Extralaryngeal soft tissue of the neck | Not involved | 84 | 56(58.3%) | 28(68.3%) | 0.273 |
|  | Involved | 53 | 40(41.7%) | 13(31.7%) |  |
| Base of tongue | Not involved | 126 | 88(91.7%) | 38(92.7%) | 1.000 |
|  | Involved | 11 | 8(8.3%) | 3(7.3%) |  |
