# Supplementary material for: Concordance between head and neck MRI and histopathology in detecting laryngeal subsite invasion among patients with laryngeal cancer
Source: Cancer Imaging. 2023 Oct 19;23:99. doi: 10.1186/s40644-023-00618-y (PMC10585883; doi:10.1186/s40644-023-00618-y)
Supplement: Supplementary file 5 — Additional file 5: Supplementary table 5. presents the sensitivity, specificity, negative predictive value, positive predictive value, and accuracy of the DWI HN-MRI sequence when predicting tumor extension into laryngeal subsites, in comparison to the findings from histopathological assessments for patients who underwent total laryngectomy. [file 40644_2023_618_MOESM5_ESM.docx]

| **Tumor extension to** | **Pathologic involvement** | **Radiologic involvement** | **Sensitivity (%)** | **Specificity (**%) | **Positive predictive value (**%) | **Negative predictive value (**%) | **Overall accuracy (**%) |
| --- | --- | --- | --- | --- | --- | --- | --- |
| Supraglottis | 76 | 86 | 86 | 80 | 97 | 40 | 85 |
| Supra and infra-hyoid epiglottis | 10 | 44 | 20 | 98 | 90 | 59 | 63 |
| Aryepiglottic folds, laryngeal aspect | 23 | 59 | 36 | 95 | 91 | 48 | 58 |
| Arytenoids | 2 | 9 | 22 | 100 | 100 | 93 | 93 |
| False vocal cords | 19 | 62 | 27 | 94 | 89 | 42 | 51 |
| True vocal cord/Glottis | 77 | 83 | 87 | 62 | 94 | 42 | 83 |
| Paraglottic space | 19 | 70 | 21 | 85 | 79 | 29 | 39 |
| Pre-eiglottic space | 15 | 39 | 36 | 98 | 93 | 69 | 73 |
| Inner cortex of thyroid cartilage | 48 | 66 | 56 | 63 | 77 | 40 | 58 |
| Anterior commissures | 8 | 49 | 12 | 96 | 75 | 51 | 53 |
| Posterior commissures | 0 | 11 | 0 | 100 | NA | 89 | 89 |
| Subglottis | 26 | 51 | 43 | 91 | 85 | 59 | 66 |
| Cricoid cartilage | 23 | 31 | 52 | 89 | 70 | 79 | 77 |
| Full-thickness thyroid cartilage | 64 | 66 | 76 | 53 | 78 | 50 | 69 |
| Extralaryngeal soft tissue of the neck | 40 | 52 | 56 | 75 | 73 | 59 | 65 |
| Base of tongue | 8 | 14 | 43 | 98 | 75 | 91 | 90 |

Supplementary table 5 presents the sensitivity, specificity, negative predictive value, positive predictive value, and accuracy of the DWI HN-MRI sequence when predicting tumor extension into laryngeal subsites, in comparison to the findings from histopathological assessments for patients who underwent total laryngectomy.
