# Supplementary material for: Concordance between head and neck MRI and histopathology in detecting laryngeal subsite invasion among patients with laryngeal cancer
Source: Cancer Imaging. 2023 Oct 19;23:99. doi: 10.1186/s40644-023-00618-y (PMC10585883; doi:10.1186/s40644-023-00618-y)
Supplement: Supplementary file 6 — Additional file 6: Supplementary table 6. presents the sensitivity, specificity, negative predictive value, positive predictive value, and accuracy of the conventional HN-MRI when predicting tumor extension into laryngeal subsites, in comparison to the findings from histopathological assessments for patients who underwent total laryngectomy. [file 40644_2023_618_MOESM6_ESM.docx]

| Tumor extension to | **Pathologic involvement** | **Radiologic involvement** | **Sensitivity (%)** | **Specificity (**%) | **Positive predictive value (**%) | **Negative predictive value (**%) | **Overall accuracy (**%) |
| --- | --- | --- | --- | --- | --- | --- | --- |
| Supraglottis | 30 | 30 | 90 | 73 | 90 | 73 | 85 |
| Supra and infra-hyoid epiglottis | 7 | 7 | 29 | 85 | 29 | 85 | 76 |
| Aryepiglottic folds, laryngeal aspect | 9 | 19 | 26 | 82 | 56 | 56 | 56 |
| Arytenoids | 1 | 3 | 0 | 97 | 0 | 92 | 90 |
| False vocal cords | 7 | 19 | 32 | 95 | 86 | 62 | 66 |
| True vocal cord/Glottis | 33 | 32 | 86 | 44 | 85 | 50 | 78 |
| Paraglottic space | 5 | 18 | 17 | 91 | 60 | 58 | 58 |
| Pre-eiglottic space | 5 | 11 | 18 | 90 | 40 | 75 | 70 |
| Inner cortex of thyroid cartilage | 14 | 16 | 69 | 88 | 79 | 81 | 80 |
| Anterior commissures | 4 | 18 | 11 | 91 | 50 | 57 | 56 |
| Posterior commissures | 1 | 6 | 0 | 97 | 0 | 85 | 82 |
| Subglottis | 20 | 17 | 76 | 70 | 65 | 81 | 73 |
| Cricoid cartilage | 12 | 13 | 54 | 82 | 58 | 79 | 73 |
| Full-thickness thyroid cartilage | 20 | 20 | 70 | 71 | 70 | 71 | 71 |
| Extralaryngeal soft tissue of the neck | 13 | 20 | 55 | 90 | 84 | 68 | 73 |
| Base of tongue | 3 | 1 | 10 | 95 | 33 | 100 | 95 |

Supplementary table 6 presents the sensitivity, specificity, negative predictive value, positive predictive value, and accuracy of the conventional HN-MRI when predicting tumor extension into laryngeal subsites, in comparison to the findings from histopathological assessments for patients who underwent total laryngectomy.
